# The role of hexokinase in cardioprotection – mechanism and potential for translation

**DOI:** 10.1111/bph.12899

**Published:** 2014-11-24

**Authors:** Andrew P Halestrap, Gonçalo C Pereira, Philippe Pasdois

**Affiliations:** ^1^ School of Biochemistry and Bristol CardioVascularUniversity of BristolBristolUK; ^2^ Present address: Department of Cell and Developmental BiologyUniversity College LondonGower StreetLondonUK; ^3^ Present address: IHU‐LIRYCInserm U1045Université de BordeauxPessacFrance

## Abstract

Mitochondrial permeability transition pore (mPTP) opening plays a critical role in cardiac reperfusion injury and its prevention is cardioprotective. Tumour cell mitochondria usually have high levels of hexokinase isoform 2 (HK2) bound to their outer mitochondrial membranes (OMM) and HK2 binding to heart mitochondria has also been implicated in resistance to reperfusion injury. HK2 dissociates from heart mitochondria during ischaemia, and the extent of this correlates with the infarct size on reperfusion. Here we review the mechanisms and regulations of HK2 binding to mitochondria and how this inhibits mPTP opening and consequent reperfusion injury. Major determinants of HK2 dissociation are the elevated glucose‐6‐phosphate concentrations and decreased pH in ischaemia. These are modulated by the myriad of signalling pathways implicated in preconditioning protocols as a result of a decrease in pre‐ischaemic glycogen content. Loss of mitochondrial HK2 during ischaemia is associated with permeabilization of the OMM to cytochrome *c*, which leads to greater reactive oxygen species production and mPTP opening during reperfusion. Potential interactions between HK2 and OMM proteins associated with mitochondrial fission (e.g. Drp1) and apoptosis (B‐cell lymphoma 2 family members) in these processes are examined. Also considered is the role of HK2 binding in stabilizing contact sites between the OMM and the inner membrane. Breakage of these during ischaemia is proposed to facilitate cytochrome *c* loss during ischaemia while increasing mPTP opening and compromising cellular bioenergetics during reperfusion. We end by highlighting the many unanswered questions and discussing the potential of modulating mitochondrial HK2 binding as a pharmacological target.

**Linked Articles:**

This article is part of a themed section on Conditioning the Heart – Pathways to Translation. To view the other articles in this section visit http://dx.doi.org/10.1111/bph.2015.172.issue‐8

**Abbreviations**
Akt: also known as PKB
AMPK: AMP‐activated PK
ANT: adenine nucleotide translocase
Bad: Bcl‐2‐associated death promoter
Bak: Bcl‐2 homologous antagonist/killer
Bax: Bcl‐2‐like protein 4
Bcl‐2: B‐cell lymphoma 2
Bcl‐xL: Bcl‐2‐extra large
CK: creatine kinase
CsA: cyclosporine A
CyP‐D: cyclophilin D
Drp1: dynamin‐related protein 1
G‐6‐P: glucose‐6‐phosphate
GSK3β: glycogen synthase kinase 3β
HK: hexokinase
I/R: ischaemia/reperfusion
IF1: ATP synthase inhibitor factor 1
IMM: inner mitochondrial membrane
IP: ischaemic preconditioning
mPTP: mitochondrial permeability transition pore
OMM: outer mitochondrial membrane
Opa‐1: optic athrophy 1
PCr: phosphocreatine
PPIase: peptidylprolyl isomerase
ROS: reactive oxygen species
SR: sarcoplasmic reticulum
T_0_: time of ischaemic rigor start
TAT‐HK2: cell‐permeable peptide of HK2 binding domain
TP: temperature preconditioning
TSPO: translocator protein of the outer membrane
VDAC: voltage‐dependent anion channel

**Tables of Links**

**Table:** 

TARGETS		
**Ion channels** *^a^*	**Enzymes** *^c^*	
Connexin 43	Akt (PKB)	GSK3β
VDAC	AMPK	PKA
**Transporters** ***^b^***	Caspases	PKCε
NHE1	Calpains	Peptidylprolyl isomerase
F‐type ATPase		

**Table:** 

LIGANDS
Carboxyatractyloside
Clotrimazole
Cyclosporine A
H2O2
Metformin

These Tables list key protein targets and ligands in this article which are hyperlinked to corresponding entries in http://www.guidetopharmacology.org, the common portal for data from the IUPHAR/BPS Guide to PHARMACOLOGY (Pawson *et al*., 2014) and are permanently archived in the Concise Guide to PHARMACOLOGY 2013/14 (*^a,b,c^*Alexander *et al*., 2013a, 2013b, 2013c).

## Introduction

It has been known for many years that both hexokinase (HK) 1 and 2 can bind to mitochondria and that this may play an important role in the regulation of HK activity and thus of glucose metabolism (Pastorino and Hoek, 2003; Wilson, 2003; Nederlof *et al*., 2014). However, evidence has emerged that binding of these enzymes to the outer mitochondrial membrane (OMM) may also decrease apoptosis mediated by members of the B‐cell lymphoma 2 (Bcl‐2) family that also bind to the OMM. Indeed, mitochondria of tumour cells exhibit both extensive HK2 binding and a resistance to opening of the mitochondrial permeability transition pore (mPTP) in the inner mitochondrial membrane (IMM) that plays a key role in both necrotic and apoptotic cell death pathways (Halestrap, 2010). Furthermore, tumour cells are very resistant to apoptotic stimuli including to agents that induce mPTP opening (Pastorino and Hoek, 2003; Robey and Hay, 2005; Mathupala *et al*., 2009). Opening of the mPTP also plays a key role in mediating ischaemia/reperfusion (I/R) injury of the heart (Baines, 2009; Halestrap, 2010; Di Lisa *et al*., 2011), and in recent years, several groups, including our own, have provided evidence that binding of HK2 to the OMM plays an important role in cardioprotection (Pasdois *et al*., 2012; Nederlof *et al*., 2014). In this paper, we will review the role of HK2 binding to mitochondria in cardioprotection, how this binding may be regulated and the mechanisms by which the binding inhibits mPTP opening and thus reperfusion injury. Finally, we will consider whether and how modulating HK2 binding to mitochondria may be translated into novel cardioprotective protocols that have clinical relevance.

## HKs

HKs catalyse the ATP‐Mg^2+^‐dependent phosphorylation of the 6‐OH of hexoses. This ensures that, following its transport across the plasma membrane, glucose is trapped in the cytosol as glucose‐6‐phosphate (G‐6‐P) and primed for further metabolism through glycolysis, the pentose phosphate pathway or glycogen synthesis. There are four isoforms of HK in mammals whose particular properties and metabolic roles have been well reviewed elsewhere (Postic *et al*., 2001; Wilson, 2003; Nederlof *et al*., 2014). Isozyme types 1–3 are quite similar in both size (monomers of about 100 kDa with fairly homologous halves) and properties (Nederlof *et al*., 2014). HK4 (also known as glucokinase) is quite different from the other HK isozymes. It is a 50 kDa monomeric protein that exhibits a much lower affinity for glucose and is not feedback inhibited by G‐6‐P (Wilson, 2003).

### Hexokinase isoforms 1 and 2 bind to the mitochondrial outer membrane

Despite its key role in glycolysis, the majority of HK1 is found associated with mitochondria where it binds to the OMM through a conserved short hydrophobic α‐helical domain in the N‐terminus (see Figure 1) that inserts into the hydrophobic core of the membrane (Xie and Wilson, 1988). Dissociation of HK1 from mitochondria can be achieved by incubation with a peptide corresponding to this domain (Gelb *et al*., 1992). The bound HK1 is thought to associate with one of the three isoforms of the voltage‐dependent anion channel (VDAC) of which VDAC1 is the most widely expressed (Pastorino and Hoek, 2008). Site‐directed mutagenesis has implicated charged residues in the transmembrane β‐barrel as well as the N‐terminus in this binding (Pastorino and Hoek, 2008; Shoshan‐Barmatz *et al*., 2009). However, details of the interaction remain uncertain as the exact topology of VDAC within the OMM is still unresolved (Colombini, 2012; Tomasello *et al*., 2013). Mitochondrial membrane fractions enriched with VDAC, HK1 and the adenine nucleotide translocase (ANT) can be purified from brain (Beutner *et al*., 1997). These are thought to represent contact sites (points of contact between the IMM and OMM) (Pastorino and Hoek, 2003; Brdiczka *et al*., 2006). A considerable body of evidence supports the proposal that these contact sites allow the ATP exported from the mitochondria via ANT (IMM) and VDAC (OMM) to be used preferentially by HK1 to phosphorylate glucose, thus linking the regulation of glycolysis with oxidative phosphorylation by mitochondria (see Wilson, 2003). A more detailed account of the structure and role of contact sites is given in the section entitled ‘The role of contact sites between the inner and OMM’, where their role in the cardioprotective effects of HK2 is considered.

**Figure 1:**
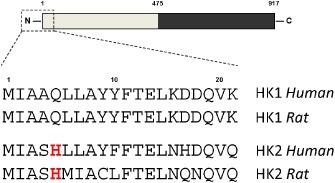
The N‐terminal sequences of HK1 and HK2 that are responsible for mitochondrial binding of the enzymes. HKs 1–3 consist of two similar halves depicted as gray and black boxes. Sequences are given for human and rat HK1 (P19367 and P05708) and HK2 (P52789 and P27881) to show the conservation of the histidine in HK2 at position 5 that is absent in HK1 and may account for the pH sensitivity of binding.

HK2 also binds to the OMM through an N‐terminal hydrophobic domain (see Figure 1) and like HK1, it can be displaced by incubation with the corresponding hydrophobic peptide (Chiara *et al*., 2008). However, unlike HK1, a significant proportion of HK2 may be present in the cytosol. The extent of mitochondrial binding depends on the prevailing metabolic conditions with high [G‐6‐P] favouring HK2 dissociation (Wilson, 2003; Pastorino and Hoek, 2008). Mitochondria of tumour cells are characterized by especially high levels of bound HK2 (Mathupala *et al*., 2009). This is thought, at least in part, to be mediated by signalling pathways that are often dysregulated in tumour cells and cause activation of the PK, Akt (Pastorino and Hoek, 2008). This has been reported to phosphorylate threonine 473 of HK2 to enhance its mitochondrial binding (Miyamoto *et al*., 2008; Roberts *et al*., 2013). In addition, it is well established that Akt can phosphorylate and inactivate glycogen synthase kinase 3β (GSK3β) which, in its active form, may phosphorylate VDAC1 on threonine 51 leading to reduced HK2 binding. Conversely, phosphorylation of VDAC1 by PKCε has been reported to increase HK2 binding (Pastorino and Hoek, 2008). Whatever the cause, increased HK2 binding to tumour cell mitochondria appears to play an important role in the resistance of tumour cells to apoptosis and displacing the bound HK2 can induce tumour cell death (Pastorino and Hoek, 2003; Chiara *et al*., 2008; Mathupala *et al*., 2009). As discussed further in the section entitled ‘The mechanisms by which HK2 binding to mitochondria inhibits mPTP opening’, this most likely relates to the role of HK2 in stabilizing contact sites and antagonizing the binding or action of pro‐apoptotic members of the Bcl‐2 family that also bind to the OMM, possibly in association with VDAC (Pastorino and Hoek, 2003; Shoshan‐Barmatz and Mizrachi, 2012).

## The central role of the mPTP in I/R injury

The mPTP is a non‐specific pore that opens in the IMM under conditions of high matrix [Ca^2+^] especially when this is accompanied by oxidative stress, elevated phosphate concentrations and decreased matrix adenine nucleotide concentrations. These are exactly the conditions that occur during reperfusion following a period of prolonged ischaemia (Halestrap, 2010). Indeed, direct measurements of mPTP opening in the Langendorff‐perfused rat heart have confirmed mPTP opening under these conditions (Griffiths and Halestrap, 1995). Importantly, pore opening is not observed in normoxic hearts or during ischaemia itself, but begins about 2 min into reperfusion (Halestrap *et al*., 1997). This corresponds to the return of the intracellular pH from the low values of ischaemia to pre‐ischaemic values (Kerr *et al*., 1999). It has been shown that the mPTP is inhibited as the pH drops below 7 (Halestrap, 1991; Bernardi *et al*., 1992), and it is thought that it is the low pH in ischaemia that keeps the pore closed despite depletion of adenine nucleotides and elevated levels of [Ca^2+^] and [Pi] (Halestrap *et al*., 2004). There is also a burst of ROS production on reperfusion, which will further stimulate mPTP opening (Halestrap and Pasdois, 2009). When open, the mPTP allows passage of any molecule <1.5 kDa, including protons, which not only prevents ATP synthesis by oxidative phosphorylation, but also causes the ATP synthase to reverse, breaking down ATP produced by healthy mitochondria and glycolysis (Di Lisa *et al*., 2011). This will compromise cardiomyocyte integrity and lead to cell death unless the mPTP closes again (Halestrap *et al*., 2004).

The molecular composition of the mPTP remains controversial and the reader is directed elsewhere for further information (Halestrap, 2009; Bernardi, 2013; Karch and Molkentin, 2014). However, one key component is well established: a mitochondrial peptidyl‐prolyl cis‐trans isomerase known as cyclophilin D (CyP‐D). Pharmacological inhibition of this with cyclosporine A (CsA) or sanglifehrin A (CsA) is cardioprotective, as is genetic knockdown of Cyp‐D (Griffiths and Halestrap, 1995; Clarke *et al*., 2002; Baines *et al*., 2005; Nakagawa *et al*., 2005). Indeed, proof of principle clinical trials have demonstrated that CsA may improve recovery of patients undergoing angioplasty following myocardial infarction (Piot *et al*., 2008; Gomez *et al*., 2009) and a large phase 3 clinical trial is underway (Kloner, 2013). Furthermore, other powerful cardioprotective strategies such as ischaemic preconditioning (IP), post‐conditioning and temperature preconditioning (TP) also exert their effects through inhibition of mPTP opening (Lim *et al*., 2007; Murphy and Steenbergen, 2007; Halestrap and Pasdois, 2009). A key question is how this inhibition is mediated. Suggested mitochondrial targets include the mitochondrial ATP‐sensitive potassium channel channels, components of the mPTP itself such as CyP‐D in the matrix and VDAC in the OMM or HK2 bound to it. Readers are referred elsewhere to review the evidence on which these claims are made (Halestrap *et al*., 2007; Hausenloy *et al*., 2009). However, here we will focus on recent data that have suggested reduced oxidative stress and increased HK2 binding play major and interconnected roles.

## Inhibition of mPTP opening by IP involves decreased oxidative stress associated with less cytochrome *c* loss during ischaemia

Use of the 2‐deoxglucose entrapment method demonstrated directly that mPTP opening *in situ* during reperfusion is less in hearts subject to IP than controls (Javadov *et al*., 2003). Furthermore, following ischaemia and reperfusion, mPTP opening by isolated mitochondria demonstrate a greater calcium sensitivity than pre‐ischaemic mitochondria, and this is associated with increased protein carbonylation, a surrogate marker for oxidative stress. Both effects are greatly attenuated by IP (Javadov *et al*., 2003; Argaud *et al*., 2004; Khaliulin *et al*., 2004; Clarke *et al*., 2008). However, no difference in the sensitivity of mPTP opening to [Ca^2+^] was observed between IP and control mitochondria when they were isolated immediately after the IP protocol. Rather, the difference emerged only after ischaemia and correlated with a decrease in the oxidative stress experienced by the mitochondria (Clarke *et al*., 2008). This was not accompanied by any translocation of PKs such as PKCε and GSK3β to mitochondria or detectable changes in phosphorylation of any mitochondrial protein (Clarke *et al*., 2008). Nor did we observe IP‐induced translocation of connexin 43 to mitochondria (Halestrap *et al*., 2007), which had previously been described by others (Rodriguez‐Sinovas *et al*., 2006; Schulz and Heusch, 2006). Taken together, our data suggested that IP does not modulate the mPTP prior to ischaemia, but rather prevents the oxidative stress that accompanies ischaemia and reperfusion and sensitizes mitochondria to mPTP opening. Our data further suggest that a key mechanism by which IP achieves this is through attenuation of cytochrome *c* release during ischaemia, and that this is regulated by HK2 binding to mitochondria.

### Extent of cytochrome *c* release during ischaemia may determine ROS levels

Several studies have demonstrated a loss of cytochrome *c* from the intermembrane space (IMS) of mitochondria during ischaemia (Lesnefsky *et al*., 1997; Borutaite *et al*., 2001; Pasdois *et al*., 2011). This leads to a decrease in ADP‐stimulated respiration and an increase in superoxide generation by mitochondria incubated under physiological conditions. Both effects were attenuated by IP and could be reversed by adding back exogenous cytochrome *c* (Pasdois *et al*., 2011; 2012). We established that loss of cytochrome *c* mediates increased ROS production through two mechanisms (Pasdois *et al*., 2011). First, oxidized cytochrome *c* acts as a scavenger of superoxide produced by the external‐facing ubiquinone site of complex 3 and its loss leads to greater release of superoxide into the IMS. Second, loss of cytochrome *c* also causes electrons to build up in complex 1 and this enhances superoxide production into the matrix. This is summarized in Figure 2. The ability of IP to prevent this cytochrome *c* loss (Pasdois *et al*., 2012) provides an attractive explanation as to how IP reduces oxidative stress and thus mPTP opening. If this explanation is correct, then the mechanisms involved become critical to understanding how IP exerts its cardioprotective effects and how this might be harnessed pharmacologically in the clinic. It should be noted that there is another important consequence of locating the site of action of IP on the OMM. It removes the need for proteins, such as kinases, that are normally resident in the cytosol, to cross the IMM and OMM in response to the IP signal, which has long been a controversial issue (see Halestrap and Pasdois, 2009).

**Figure 2:**
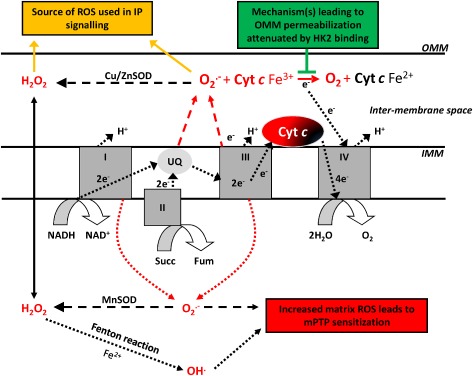
Scheme illustrating how cytochrome *c* acts as a potent scavenger of superoxide whose loss may increase both matrix and extramitochondrial ROS. Cytochrome c loss restricts electron flow leading to an increase in the reduction state of the pool of ubiquinone, complex I and complex II. Moreover, the rate of anion superoxide oxidation by the pool of oxidized cytochrome *c* decreases secondary to its leak in the cytosol. These events lead to an excess of ROS production in the matrix and the IMS, thus sensitizing mPTP to calcium.

## Mechanisms involved in cytochrome *c* loss during ischaemia

Cytochrome *c* release during ischaemia involves a specific permeabilization of the OMM that is prevented by IP. However, the molecular mechanisms involved are not fully understood.

### Role of Bcl‐2 family members

In apoptosis, the mechanism of cytochrome *c* release involves a specific permeabilization of the OMM mediated by pro‐apototic members of the Bcl‐2 family such as Bcl‐2‐like protein 4 (Bax), Bcl‐2 homologous antagonist/killer (Bak) and Bcl‐2‐associated death promoter (Bad), whose actions are opposed by the anti‐apoptotic members of the family such as Bcl‐2 and Bcl‐2‐extra large (Bcl‐xL) (Tait and Green, 2010). In terminally differentiated cells such as myocytes, some of these proteins such as Bak and Bcl‐xL are normally present in the OMM, and it is a change in their ratio, mediated by various mechanisms such as phosphorylation, proteolysis and translocation, that causes permeabilization (see Soriano and Scorrano, 2010; Tait and Green, 2010; Martinou and Youle, 2011). Furthermore, hearts from mice in which both Bax and Bak have been knocked out are greatly protected from reperfusion injury and no further protection is provided by CyP‐D knockout, implying that the protection is mediated via prevention of mPTP opening (Whelan *et al*., 2012). Although we were able to confirm the presence of Bax and Bak in heart mitochondria, we were unable to detect an increase following ischaemia. Smeele *et al*. (2011) were also unable to detect a change in Bax under these conditions. Neither were we able to detect changes in other pro‐apoptotic protein such Bad and BH3 interacting‐domain death agonist (Pasdois *et al*., 2011). However, we did observe a decrease in the anti‐apoptotic protein Bcl‐xL. This protein works in opposition to the pro‐apoptotic Bax and Bad and its decrease in ischaemia is a promising candidate for mediating the increased OMM permeability. Indeed, adenovirus‐mediated Bcl‐xL gene transfer has been reported to protect hearts from I/R injury (Huang *et al*., 2003). However, we were unable to observe any effect of IP on either the loss of Bcl‐xL following ischaemia, or on the expression levels of other Bcl‐2 family members (Pasdois *et al*., 2012). This suggests that other factors must be involved in regulating cytochrome *c* release and HK2 is a strong candidate for this role.

### A role for HK2 in regulating cytochrome *c* release

The group of Zuurbier was first to implicate HK2 binding to mitochondria in cardioprotection by demonstrating that the amount of HK2 bound to subsequently isolated mitochondria increased following IP or morphine treatment of hearts, without any change in its phosphorylation or in HK1 binding (Zuurbier *et al*., 2005; Gurel *et al*., 2009). Subsequently, they demonstrated a progressive loss of bound HK2 during ischaemia (Gurel *et al*., 2009). Although no difference in bound HK2 was found between control and IP hearts after 35 min ischaemia in these studies, the effect of IP re‐emerged as binding increased again on reperfusion. Our own studies confirmed a substantial loss of mitochondrial HK2 after 30 min global ischaemia, but found this loss to be largely prevented by IP (Pasdois *et al*., 2012). Again, no changes were observed in HK1 binding. Our data are entirely consistent with the proposal that IP prevents loss of cytochrome *c* during ischaemia by maintaining HK2 binding to mitochondria and imply that dissociation of mitochondrial HK2 should attenuate cardioprotection by IP. Such an effect was confirmed by Zuurbier's laboratory (Smeele *et al*., 2011) using a cell‐permeable peptide of HK2 binding domain (TAT‐HK2) whose sequence corresponds to the mitochondrial binding motif of human HK2 and which others had shown causes HK2 dissociation from mitochondria (Gelb *et al*., 1992; Chiara *et al*., 2008). Perfusion of hearts with this TAT‐HK2 peptide significantly decreased mitochondrial HK2 levels without affecting baseline cardiac function. However, when perfused prior to ischaemia it dramatically increased I/R injury and prevented the protective effects of IP (Smeele *et al*., 2011). Similar studies in this laboratory confirmed that TAT‐HK2 peptide increases I/R injury, but suggested that rather than directly dissociating HK2 from myocyte mitochondria, the peptide was having an indirect effect on heart function through vasoconstriction of the coronary circulation and consequent hypoxia (Pasdois *et al*., 2013). Although Zuurbier and colleagues have challenged these conclusions (Nederlof *et al*., 2013), we sought other means to modulate mitochondrial HK2 binding that could be used to investigate its role in cardioprotection (Pasdois *et al*., 2012).

### The extent of HK2 dissociation from mitochondria during ischaemia correlates with the severity of reperfusion injury

In our studies, we modulated HK2 binding by changing cytosolic [G‐6‐P] through metabolic interventions that target glycogen metabolism and glycolysis. A very strong negative correlation was found between the HK activity measured in mitochondria isolated at the end of ischaemia and the infarct size after 2 h reperfusion. The change in HK activity was confirmed to reflect changes in HK2 rather than HK1 binding (Pasdois *et al*., 2012). Data are shown in Figure 3, and the underlying mechanisms discussed more fully in the section entitled ‘G‐6‐P and pH modulate HK2 binding – a link between IP and metabolism’. Interestingly, recent measurements of cytosolic and particulate HK activity bound to mitochondria from control and diabetic hearts revealed that IP fails to modulate the redistribution of HK2 during ischaemia in diabetic hearts (Gurel *et al*., 2013). This may explain the impaired ability of IP to protect the diabetic heart from reperfusion injury (Kersten *et al*., 2000; Tsang *et al*., 2005).

**Figure 3:**
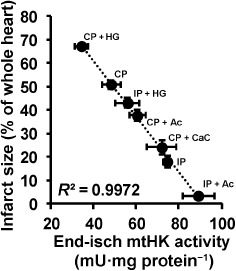
The activity of HK bound to mitochondria at the end of 30 min ischaemia is inversely correlated with infarct size after 2 h reperfusion. For greater accuracy the amount of mitochondrial‐bound HK2 at the end of ischaemia was determined by measuring enzyme activity, but it was confirmed that only HK2 and not HK1 is lost during ischaemia. The pretreatments used to modify HK2 binding at the end of ischaemia were: control (CP), ischaemic preconditioned (IP) and perfusion with acetate (Ac), high [Ca ^2+^] (CaC) or high glucose (HG) with or without IP. Data are taken from Pasdois *et al*. (2012). mtHK, mitochondrial HK.

## The mechanisms involved in regulation of HK2 binding to mitochondria

### VDAC and HK2 phosphorylation are not involved

There is strong evidence that PKCε plays an important role in the signalling pathway for IP (Budas *et al*., 2007), and it has been reported that in the heart, PKCε can translocate to the mitochondria where it binds to and phosphorylates VDAC1 to increase HK2 binding (Baines *et al*., 2003). However, we were unable to demonstrate either PKCε translocation to mitochondria or a change in the phosphorylation state of any VDAC isoform in response to IP (Clarke *et al*., 2008). Conversely, Pastorino *et al*. have reported that GSK3β can phosphorylate VDAC1 in tumour cells, reducing mitochondrial HK2 binding and so making the cells more sensitive to chemotherapeutic agents (Pastorino *et al*., 2005). Akt phosphorylates and inactivates GSK3β explaining how activation of Akt may enhance HK2 binding to mitochondria while its inhibition promotes dissociation (Pastorino and Hoek, 2008; Neary and Pastorino, 2013). These data suggest that the IP could increase HK2 binding to heart mitochondria through a decrease in GSK3β‐dependent phosphorylation of VDAC caused by Akt whose activation has been strongly implicated in mediating IP (Hausenloy and Yellon, 2006; Yang *et al*., 2010). Indeed, it has been proposed that the mechanisms of many cardioprotective regimes converge to phosphorylate and inhibit GSK3β whose activity promotes mPTP opening (Juhaszova *et al*., 2004; 2009). However, others have reported that neither Akt stimulation with insulin nor genetic ablation of VDAC affects cell death induction by HK2 dissociation (Chiara *et al*., 2008). An alternative means to link the Akt pathway to HK2 binding would be through phosphorylation of HK2 (on Thr^473^) by Akt itself, which is reported to enhance HK2 binding to mitochondria in cardiomycoytes and reduce its sensitivity to dissociation by increased concentrations of G‐6‐P (Miyamoto *et al*., 2008; Roberts *et al*., 2013).

Although activation of Akt may appear to be an attractive link between the IP stimulus and cardioprotection mediated by HK2 binding to mitochondria, it is unlikely to be the major mechanism for two reasons. First, insulin, a potent activator of the Akt pathway, is not cardioprotective unless added during reperfusion (Juhaszova *et al*., 2004). Indeed, pre‐ischaemic treatment of hearts with insulin in place of the IP protocol causes Akt and GSK3β phosphorylation to increase several fold, yet no cardioprotection is observed (Clarke *et al*., 2008), and such treatment may even suppress cardioprotection by IP (Fullmer *et al*., 2013). These observations can be explained through effects on tissue glycogen levels and consequent effects on HK2 binding as will be described later (‘G‐6‐P and pH modulate HK2 binding – a link between IP and metabolism’). Second, we were unable to detect any changes in the amount of Akt or GSK3β detected in a highly purified mitochondria following the IP protocol. Nor were we able to detect significant changes in phosphorylation of any mitochondrial proteins (including VDAC and HK2) (Clarke *et al*., 2008).

### G‐6‐P and pH modulate HK2 binding – a link between IP and metabolism

HK1 has been reported to dissociate from isolated heart mitochondria when G‐6‐P or ATP are added, an effect prevented or reversed by the presence of Mg^2+^ or phosphate (Aubert‐Foucher *et al*., 1984) and G‐6‐P has also been shown to cause HK2 dissociation from tumour cell mitochondria (Pastorino *et al*., 2002). Thus, another mechanism by which IP might prevent HK2 dissociation during ischaemia would be through decreased [G‐6‐P]. Such a decrease has been demonstrated previously by others (Murry *et al*., 1990; Jennings *et al*., 2001) and recently confirmed in this laboratory (Pasdois *et al*., 2012). G‐6‐P is formed during ischaemia by glycogen breakdown, and it has been shown previously that one factor that determines the extent of reperfusion injury is the pre‐ischaemic glycogen content of the heart (Finegan *et al*., 1995; Cross *et al*., 1996; King and Opie, 1996; McNulty *et al*., 1996). Previously, it was argued that this effect was a consequence of less glycolysis and lactic acid accumulation during ischaemia leading to a smaller decrease in pH as observed in IP ischaemic hearts mediated. This, in turn would attenuate the sodium and calcium loading of the cell mediated by the sodium proton antiporter (NHE1) and the sodium calcium antiporter respectively. The reduced calcium overload would then lead to less mPTP opening on reperfusion as observed (Halestrap *et al*., 1998). However, attenuation of G‐6‐P induced dissociation of mitochondrial HK2 during ischaemia provides an attractive additional mechanism by which glycogen depletion prior to ischaemia may be cardioprotective. In support of this, we used saponin‐permeabilized heart muscle fibres to demonstrate that increasing [G‐6‐P] can dissociate HK2 from mitochondria, but only when accompanied by a decrease in pH such as that which occurs during ischaemia. Indeed, optimal dissociation was observed at pH 6.3, which is similar to the intracellular pH determined at the end of a 30 min ischaemia (Pasdois *et al*., 2012). We have also confirmed that incubation of isolated rat heart mitochondria with G‐6‐P at pH 6.3 leads to dissociation of bound HK2 (G.C. Pereira and A.P. Halestrap, unpubl. data). It is of interest that HK1 does not dissociate under these conditions and neither does HK1 dissociate from mitochondria during ischaemia (Gurel *et al*., 2009; Pasdois *et al*., 2011). We have suggested that this reflects the lack of a histidine in the N‐terminal helix of HK1 that binds it to the OMM membrane as shown in Figure 1 (Pasdois *et al*., 2012).

In order to confirm that the link between cardioprotection and pre‐ischaemic glycogen content can be explained through differences in end‐ischaemic [G‐6‐P] and pH on mitochondrial HK2 binding, we perfused hearts under a variety of conditions previously reported by King and Opie (1996) to modulate glycogen content prior to ischaemia and rates of glycolysis during ischaemia (Pasdois *et al*., 2012). As a surrogate marker of glucose metabolism during ischaemia we determined the time at which ischaemic rigor starts (T_0_) as this has been shown to occur when glycolysis stops (Kingsley *et al*., 1991). We demonstrated that the smaller T_0_, the better the cardioprotection observed, which is consistent with cardioprotection being associated with an early inhibition of glycolysis, and hence decreased [G‐6‐P] and a smaller drop in pH_i_ leading to reduced dissociation of HK2 from mitochondria. However, even more impressive was the strong inverse correlation between infarct size after 2 h reperfusion with end‐ischaemic mitochondrial HK activity (Figure 3). These data confirm the extremely strong association between mitochondrial HK2 binding at the end of ischaemia and heart recovery during the reperfusion period, and suggest that pre‐ischaemic glycogen content and rates of glycolysis during ischaemia play an important role in modulating HK2 binding through changes in [G‐6‐P] and pH. As a range of signalling pathways can affect both glycogen metabolism and glycolysis, this may provide some insight into why such a variety of signalling pathways have been implicated in preconditioning (Halestrap *et al*., 2007; Murphy and Steenbergen, 2008; Yang *et al*., 2010).

### Pre‐ischaemic glycogen content alone does not determine the severity of I/R injury

It is important to recognize, as have others (Asimakis, 1996; King and Opie, 1996; Schaefer and Ramasamy, 1997), that pre‐ischaemic glycogen content alone does not determine the extent of I/R injury. For example, pre‐ischaemic glycogen content increases following starvation to a level similar to that seen with pre‐ischaemic insulin treatment, yet starvation is associated with cardioprotection whereas pre‐ischaemic insulin treatment leads to greater injury (Schaefer and Ramasamy, 1997). Similarly, TP is highly cardioprotective, and yet is associated with ischaemic contracture taking longer to develop, which is indicative of higher glycogen levels (Khaliulin *et al*., 2007). Conversely, glucagon treatment of rat hearts is associated with a decrease in pre‐ischaemic glycogen content and reduced lactate production during ischaemia, and yet no cardioprotection is observed and no decrease in G‐6‐P concentrations detected (Vander Heide *et al*., 1996a). These observations highlight that factors other than pre‐ischaemic glycogen content may regulate the rate of glycolysis, and thus the pH decrease during ischaemia. Indeed in a porcine model of I/R injury, rates of glycolysis and lactate accumulation during ischaemia were a better predictor of injury than either ATP depletion or G‐6‐P levels, but they did not always correlate with pre‐ischaemic glycogen content (Vogt *et al*., 2002a, 2002b). It has also been demonstrated that too little glycogen can actually exacerbate I/R injury (King and Opie, 1996), although only haemodynamic function rather than an indicator of necrotic injury such infarct size was determined in these studies. Nevertheless, it is possible that gross depletion of glycogen prior to ischaemia not only speeds up the loss of ATP and loss of bioenergetic competence, but also causes a greatly reduced drop in pH during ischaemia that may be insufficient to inhibit mPTP opening.

### Other mechanism involved in modulating HK2 binding and cardioprotection in response to preconditioning

There are likely to be important additional mechanisms operating in parallel to these metabolically induced changes in HK2 binding that may also be involved in mediating cardioprotection in response to different preconditioning protocols. For example, when added before ischaemia, insulin, which stimulates glycogen synthesis, is not cardioprotective and may even exacerbate injury and prevent protection by IP (Clarke *et al*., 2008; Fullmer *et al*., 2013). Yet, when present during reperfusion, the same signalling pathway involving Akt phosphorylation and inhibition of GSK3β is cardioprotective (Hausenloy and Yellon, 2006; Murphy and Steenbergen, 2008; Yang *et al*., 2010). As noted earlier, in tumour cells, Akt activation enhances HK2 binding to mitochondria, and Akt inhibition causes HK2 dissociation probably via inhibition of GSK3β (Pastorino *et al*., 2005; Neary and Pastorino, 2013). Thus, the cardioprotective effects of Akt activation and GSK3β inhibition during reperfusion may be important in facilitating the rebinding of HK2 to the mitochondria, thereby contributing towards the protection against mPTP opening. Indeed, Gurel *et al*. (2009) have reported that HK2 binding to mitochondria, which decreases following ischaemia, increases again on reperfusion and more so in IP hearts.

## The mechanisms by which HK2 binding to mitochondria inhibits mPTP opening

The correlation between end‐ischaemic mitochondrial HK2 and cardioprotection during reperfusion is consistent with HK2 binding to the OMM both reducing its permeability to cytochrome *c* and making the mitochondria less vulnerable to mPTP opening. This might occur if bound mitochondrial HK2 interacted directly with members of the Bcl‐2 family, perhaps in association with VDAC, to prevent them forming an active permeability pathway for cytochrome *c* in the OMM. However, this is not supported by experiments performed with permeabilized fibres isolated from end‐ischaemic hearts (Pasdois *et al*., 2012). Here the prevention of cytochrome *c* release provided by IP could not be reversed by incubation with G‐6‐P at pH 6.3 to dissociate the bound HK2. An attractive alternative hypothesis would be that HK2 binding has effects on mitochondrial morphology involving changes in the interaction between the IMM and OMM that modulate both OMM permeability to cytochrome *c* and MPTP opening.

### Mitochondrial morphology and cytochrome *c* release

There is increasing evidence that cytochrome *c* release during apoptosis involves changes in mitochondrial cristae morphology allowing the cytochrome *c* within the cristae to gain access to the permeabilized OMM (Martinou and Youle, 2011). A major factor in the control of mitochondrial cristae morphology is optic athrophy 1 (Opa‐1), which is thought to tighten the cristae junction and prevents cytochrome *c* release while fission in apoptosis is induced by mitochondrial recruitment of dynamin‐related protein 1 (Drp1), a dynamin‐related GTPase (Martinou and Youle, 2011; Hall *et al*., 2014). Interestingly, mitochondria have also been reported to undergo fission during ischaemia, and inhibiting Drp1 activity with the mitochondrial division inhibitor 1 (Ong *et al*., 2010; Disatnik *et al*., 2013; Gao *et al*., 2013; Sharp *et al*., 2014) or transgenic expression of a dominant negative form of Drp1 (Zepeda *et al*., 2014) leads to reduced infarct size following ischaemia and reperfusion. This is accompanied by preserved mitochondrial morphology, reduced mitochondrial ROS production and improved haemodynamic function (Sharp *et al*., 2014). These data are consistent with Drp1 recruitment during ischaemia leading to the opening of cristae junctions, allowing cytochrome *c* to be released through the permeabilized OMM. However, we have been unable to demonstrate changes in either Drp1 or Opa‐1 content of mitochondria following ischaemia (P. Pasdois and A.P. Halestrap unpubl. data), suggesting that some other mechanism(s) may operate. An attractive candidate is the contact sites that are known to exist between IMM and OMM that are associated with HK2 binding (Brdiczka *et al*., 2006).

### The role of contact sites between the IMM and OMM

Freeze fracture of mitochondria revealed places where the IMM and OMM interact closely, and that these ‘contact sites’ are very dynamic structures, which are affected by the metabolic status of mitochondria (Knoll and Brdiczka, 1983; Brdiczka, 1991). Contact sites are thought to be held in place by a complex of proteins comprising VDAC, ANT, and when present, HK or creatine kinase (CK) which co‐purify during membrane sub‐fractionation of mitochondria (Knoll and Brdiczka, 1983; Beutner *et al*., 1998). Contact sites were originally thought to function primarily to enhance the transfer of ATP from the matrix to the cytosol either directly or via creatine phosphate (Brdiczka, 1991) and only subsequently were implicated in the regulation of OMM permeability and the mPTP (Brdiczka *et al*., 2006).

In isolated liver mitochondria, which lack both CK and bound HK2 to stabilize contact sites, cytochrome *c* release can be inhibited by adding dextran to mimic the presence of cytosolic proteins that usually maintain osmotic equilibrium across the OMM (Doran and Halestrap, 2000). In the absence of such an osmotic support, the IMS can swell causing rupture of the relatively weak contact sites, opening of cristae junctions and thus access of cytochrome *c* within the cristae to the permeation pathways in the OMM. The presence of dextran also reduces the sensitivity of the mPTP towards [Ca^2+^] (Doran and Halestrap, 2000) suggesting that contact sites may have the additional effect of inhibiting mPTP opening directly. Indeed, stabilizing contact sites by adding exogenous HK1 also attenuates mPTP opening, but only if the N‐terminal tail involved in OMM binding is present (Azoulay‐Zohar *et al*., 2004). Conversely, increasing matrix volume sensitizes mPTP opening to [Ca^2+^], perhaps by unfolding the cristae and disrupting the cristae junctions associated with contact sites (Connern and Halestrap, 1996). Thus, it may be significant that mitochondria isolated from ischaemic hearts have an increased matrix volume (Lim *et al*., 2002). Modulation of contact sites may also provide the mechanism through which ligands of peripheral membrane benzodiazepine receptor [also known as translocator protein of the outer membrane (TSPO) ] act to inhibit mPTP opening and induce cardioprotection (Schaller *et al*., 2010). Direct regulation of the mPTP by TSPO has now been ruled out by the demonstration that mPTP opening in mitochondria from mice in which TSPO has been conditionally knocked out is no different from control mitochondria (Sileikyte *et al*., 2014).

Cristae structure can also be modulated by dimerization of F_1_F_o_ ATP synthase such as occurs in response to binding of the ATPase inhibitor protein, ATP synthase inhibitor factor 1 (IF1) (Campanella *et al*., 2008), and thus it may be significant that IP has been reported to inhibit F_1_F_o_ ATPase activity during ischaemia (Ala‐Rami *et al*., 2003). In addition, Cyp‐D, a component of the mPTP, can also bind to and modulate the ATPase activity of the F_1_F_o_ ATP synthase (Giorgio *et al*., 2009), thus providing another potential link between contact sites, cristae structure and mPTP opening. Furthermore, several groups have implicated the F_1_F_o_ ATP synthase directly in mPTP formation although there is no consensus as to how this is achieved (Bernardi, 2013; Giorgio *et al*., 2013; Bonora *et al*., 2014; Karch and Molkentin, 2014). Another factor that can modulate both contact site formation and mPTP opening is carboxyatractyloside that traps the ANT in its ‘c’ conformation (Doran and Halestrap, 2000; Brdiczka *et al*., 2006).

Since contact sites appear to be stabilized by HK2 binding (Kottke *et al*., 1991; Beutner *et al*., 1997), it would be reasonable to assume that the loss of HK2 during ischaemia leads to contact site breakage and thus would account for the observed release of cytochrome *c* and sensitization of mPTP opening to [Ca^2+^]. Consistent with this is the observation that knockout of CK, whose presence further stabilizes contact sites (Speer *et al*., 2005), makes hearts more sensitive to I/R injury (Spindler *et al*., 2004). Interestingly, liver mitochondria from mice genetically modified to express mitochondrial CK (which is normally absent) demonstrated a threefold increase in the number of contact sites as observed by electron microscopy (Speer *et al*., 2005), and were more resistant to mPTP opening by calcium (Dolder *et al*., 2003).

### Contact sites are broken in ischaemia, but stabilized if HK2 dissociation is prevented

As noted earlier, contact sites are thought to play an important role in transporting ATP generated within the mitochondria to the cytosol as phosphocreatine (PCr) (Brdiczka *et al*., 2006). Using normoxic saponin‐permeabilized heart muscle fibres, we showed that dissociation of HK2 from mitochondria by treatment with G‐6‐P at pH 6.3 led to decreased rates of extramitochondrial PCr synthesis consistent with disruption of contact sites (Pasdois *et al*., 2012). It is of interest that the ability of the bound HK2 to enhance PCr synthesis under these conditions required the presence of glucose to enable HK2 activity, while others have reported that the protective effect of mitochondrial HK binding on cell survival also requires the presence of glucose (Gottlob *et al*., 2001). The reason for this glucose requirement is not known, but suggests that the conformation of HK2 may be important in maintaining the contact site in a state that mediates the PCr shuttle.

In the perfused heart, there is good evidence for an impairment of the PCr shuttle following ischaemia (Rauch *et al*., 1994), which is improved following IP (Laclau *et al*., 2001). This is consistent with contact site breakage during ischaemia and its prevention by IP. We provided direct evidence for this by freeze‐clamping hearts at various time points over the first 90 s of reperfusion following 30 min ischaemia, and determining their PCr and ATP content (Pasdois *et al*., 2012). As predicted, we showed that the recovery of PCr was greatest in hearts with the highest mitochondrial HK2 binding. Furthermore, this was accompanied by less end‐diastolic dysfunction during the onset of reperfusion (Pasdois *et al*., 2012). To explain this, we proposed that enhanced HK2 binding at the end of ischaemia maintains functional contact sites that allow faster rates of PCr synthesis in the cytosol and thus more rapid regeneration of the cytosolic ATP. This is required to drive sarcoplasmic reticulum (SR) uptake of the calcium that accumulates in the cytosol during ischaemia and so supports a more rapid decline in the end‐diastolic pressure. None of these effects are likely to be secondary to changes in opening of the mPTP, which stays closed for the first 2 min of reperfusion (Halestrap *et al*., 2004). This was confirmed by demonstrating that the presence of 0.2 μM CsA to inhibit mPTP opening during ischaemia was without effect on either PCr or haemodynamic recovery during the first 90 s of reperfusion (Pasdois *et al*., 2012).

## HK2 binding to mitochondria may explain the cardioprotective effect of many different preconditioning protocols

The very strong inverse correlation between the extent of HK2 binding to mitochondria at the end of ischaemia with the extent of damage (infarct size) following reperfusion (Figure 3) suggests that HK2 plays a critical role in cardioprotection (Pasdois *et al*., 2012). An attractive feature of this proposal is that it may also explain how a diverse range of known cardioprotective regimes and signalling pathways can produce the same final outcome of stabilizing contact sites, reducing OMM permeabilization and inhibiting mPTP opening on reperfusion. Thus, metabolic interventions that are cardioprotective may modulate HK2 binding to mitochondria through changes in G‐6‐P levels and pH_i_ that are secondary to alterations in glycogen metabolism. The latter can be regulated by a variety of kinase cascades including those implicated in IP and TP such as PKC, PKA, Akt and GSK3β pathways (Hausenloy and Yellon, 2006; Khaliulin *et al*., 2010; Yang *et al*., 2010). In some contexts, these kinases may also influence HK2 binding through other means including phosphorylation of OMM proteins such as VDAC or HK2 itself (Jennings *et al*., 1991; Miyamoto *et al*., 2008; Pastorino and Hoek, 2008; Kerner *et al*., 2012; Roberts *et al*., 2013). Other targets for regulation are members of the Bcl‐2 family whose activity can also be regulated by phosphorylation, as well as by proteolysis and translocation (Tait and Green, 2010).

It could be argued that the increased HK2 binding to mitochondria at the end of ischaemia is a consequence of cardioprotection rather than the causative mechanism. However, there are two strong arguments against this. First, hearts from HK2^+/−^ mice have reduced HK2 content and are more sensitive to I/R injury (Wu *et al*., 2011). Second, a plausible model can be proposed that is able to explain how enhancing HK2 binding to mitochondria at the end of ischaemia may mediate many of the observed effects associated with preconditioning that lead to cardioprotection. This model, illustrated in Figure 4 and described in detail below, also highlights areas where further research is required.

**Figure 4:**
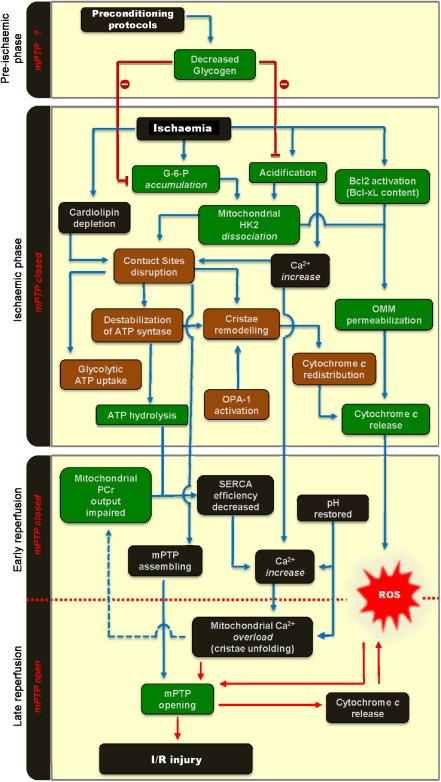
A scheme illustrating how ischaemia may lead to loss of cytochrome *c* during ischaemia and a progressive increase in [Ca ^2+^], ROS production and mPTP opening during reperfusion. This is a modified version of the scheme presented in Pasdois *et al*. (2012). It is suggested that a primary mechanism by which preconditioning protocols act to prevent HK2 loss during ischaemia is via depletion of glycogen and thus reduce glycolysis, acifidification and G‐6‐P levels during ischaemia. However, this does not exclude additional sites of action mediated via other signalling pathways that modulate mitochondrial morphology through alternative mechanisms such as phosphorylation and/or sumoylation of the fission protein Drp1. Green boxes indicate events that we have previously reported to occur during I/R injury and/or preconditioning. Black boxes represent known events reported by others. Brown boxes are hypothetical events that may occur during ischaemia, but need experimental support.

### A model that accounts for the cardioprotective effects of HK2 binding to mitochondria

We propose that during ischaemia, the breakdown of glycogen leads to increased [G‐6‐P], rates of glycolysis and lactic acid production and thus to a decrease in pH_i_. Together, these lead to HK2 dissociation from mitochondria and this, combined with the increase in [Ca^2+^] that develops during ischaemia, will destabilize contact sites, opening up the cristae and enabling cytochrome *c* to be released across the OMM. The actual permeation pathway for cytochrome *c* remains uncertain, but is probably provided by channels formed by unmasking pro‐apoptotic members of the Bcl‐2 family such as Bax and Bak already in the OMM (Pasdois *et al*., 2012). We suggest that this unmasking is caused by the observed loss of Bcl‐xL (Pasdois *et al*., 2011) which might be mediated by proteolytic degradation involving caspases or calpains, inhibitors of which have been shown to be cardioprotective (Inserte *et al*., 2012). Cytochrome *c* loss will also lead to greater ROS production (Pasdois *et al*., 2011), at least in part accounting for the oxidative stress observed following I/R. The combination of oxidative stress and raised [Ca^2+^] will then cause opening of the mPTP (Halestrap, 2010; Pasdois *et al*., 2012). This mechanism provides an explanation as to why hearts from mice in which Bax and Bak have been knocked out show reduced necrotic damage (infarct size) following ischaemia and reperfusion (Whelan *et al*., 2012). Conversely, hearts from mice deficient in CK, which stabilizes contact sites, show increased vulnerability to reperfusion injury (Spindler *et al*., 2004; Whelan *et al*., 2012).

In addition to effects on cytochrome *c* release, breakage of contact sites may also enhance the sensitivity of the mPTP to [Ca^2+^] directly (Doran and Halestrap, 2000) by disrupting the observed inhibitory effects of the OMM on mPTP opening (Chiara *et al*., 2008; Sileikyte *et al*., 2011; 2014). Conversely, mPTP opening itself induces cytochrome *c* loss by matrix swelling and OMM rupture, and this will further increase ROS production, potentially leading to a cascade of mPTP opening in adjacent mitochondria as reperfusion continues (Zorov *et al*., 2006; Halestrap and Pasdois, 2009). Another important mechanism by which HK2 dissociation from mitochondria and contact site breakage during ischaemia may increase damage to the heart during reperfusion is through inhibition of ATP channelling from the mitochondria to the cytoplasm. This will lead to impaired reuptake of Ca^2+^ into the SR as has been observed in CK‐deficient mice (Spindler *et al*., 2004). If, during early reperfusion, the SR is less able to take up the cytosolic calcium that has accumulated during ischaemia, more calcium will be accumulated by mitochondria and this, together with the oxidative stress caused by loss of cytochrome *c*, will induce mPTP opening (Halestrap, 2010; Pasdois *et al*., 2012).

Breakage of contact sites during ischaemia may also play a role in accelerating the rate of ATP hydrolysis by mitochondria during ischaemia. During ischaemia, the F_1_F_o_ ATP synthase works in reverse, hydrolysing ATP to maintain the proton motive force (Campanella *et al*., 2009). The resulting loss of ATP perturbs ionic homeostasis and is accompanied by net loss of adenine nucleotides from the heart that is one cause of the reduced haemodynamic function of hearts (stunning) following ischaemia (Halestrap and Pasdois, 2009). In addition, adenine nucleotides are lost from the mitochondrial matrix, which will further sensitize the mitochondria to mPTP opening (Griffiths and Halestrap, 1995). Indeed, prevention of ATP breakdown during ischaemia using BMS‐199264, a selective inhibitor of the hydrolytic activity of the F_1_F_o_ ATP synthase, was reported to reduce infarct size on reperfusion as would be predicted (Grover *et al*., 2004). Preconditioning also slows the breakdown of ATP during ischaemia and it was originally proposed that this was mediated by increased binding of the ATPase inhibitor protein, IF1 (Murry *et al*., 1990; Jennings *et al*., 1991; Vuorinen *et al*., 1995), although this has not been confirmed by others using direct measurement of ATPase activity (Vander Heide *et al*., 1996b; Green *et al*., 1998). However, the stabilization of contacts sites by IP may provide an attractive alternative explanation for the slower loss of ATP during ischaemia as it has been reported that HK1 binding to VDAC decreases its conductance which might allow it to transport PCr (MWt 211), but not ATP (MWt 507) (Azoulay‐Zohar *et al*., 2004). If HK2 binding exerts a similar effect, maintaining its presence during ischaemia by preconditioning will reduce ATP access to the mitochondria and hence support lower rates of ATP hydrolysis as observed, while still allowing free permeation of PCr to the cytosol on reperfusion. Support for this proposal comes from the observation that addition of HK2 to isolated liver and heart mitochondria decreased the rate of transport of fluorescently labelled ATP into mitochondria in a manner similar to that induced by Koenig's polyanion, a known VDAC inhibitor (Perevoshchikova *et al*., 2010).

## Outstanding issues relating to the role of HK2 in preconditioning

There are several features of the model summarized in Figure 4 that still require experimental confirmation and elucidation of the underlying molecular mechanisms. For example, it will be important to demonstrate stabilization of contact sites during ischaemia directly by analysing the IMM and OMM contacts and cristae morphology of mitochondria from control and preconditioned hearts before and after ischaemia using electron microscopy with three‐dimensional tomography. Work is underway in the laboratory to this end. Another uncertainty is the protein binding partner for HK2 in the OMM; so too is the mechanism by which this stabilizes contact sites. Also, to be established is the exact role played by members of the Bcl‐2 family, CK and proteins involved in mitochondrial fusion and fission such as Drp1 in stabilizing or destabilizing contact sites and modulating cristae conformation. How HK2 interacts with these proteins is also unknown. Establishing the relative contribution of each of these effects and the underlying mechanisms will be challenging. Furthermore, it is probable that some of the cardioprotective effects of IP are independent of HK2 binding to mitochondria and contact site stabilization. Nevertheless, the evidence that contact sites play an important role in cardioprotection is strong as either partial knockout of HK2 or total knockout of mitochondrial CK, that are both involved in stabilizing contact sites, enhances reperfusion injury (Spindler *et al*., 2004; Smeele *et al*., 2011).

## Potential for translation to the clinic

Although there are drugs that have been reported to dissociate HK2 from mitochondria such as clotrimazole (Penso and Beitner, 1998) and oroxylin A (Wei *et al*., 2013), there are currently no known drugs that directly enhance binding. The widely prescribed antidiabetic drug, metformin, has been reported to increase HK2 translocation to mitochondria in diabetic hearts (Da Silva *et al*., 2012) and does have cardioprotective effects in both diabetic and non‐diabetic animals (Bhamra *et al*., 2008; Calvert *et al*., 2008; Solskov *et al*., 2008; Whittington *et al*., 2013). However, the underlying mechanism is unclear and is more likely to be indirect through activation of AMPK (Nederlof *et al*., 2014). Up‐regulating HK2 expression in the heart provides another potential means of increasing mitochondrial HK2 binding, and this may be possible by modulating levels of the microRNAs miR‐155 and miR‐143. These have been reported to up‐regulate and down‐regulate HK2 expression respectively (Jiang *et al*., 2012). Nevertheless, in cardiac surgery and angioplasty, these approaches are unlikely to be as suitable or effective as established cardioprotective regimes such as IP (direct and remote), TP, and ischaemic post‐conditioning or their pharmacological mimics (Hausenloy and Yellon, 2011; Khaliulin *et al*., 2011; Hausenloy *et al*., 2012; Lim and Hausenloy, 2012; Ovize *et al*., 2013). Of these, remote preconditioning and post‐conditioning are currently the most amenable for use in the clinic, but no data are available on the effects of these treatments on mitochondrial HK2 binding. TP can be mimicked by consecutive activation of PKA and PKC prior to the ischaemic episode, and this intervention, which is both clinically feasible and more cardioprotective than IP, does decrease the pre‐ischaemic glycogen content of the heart suggesting that it probably maintains HK2 binding to mitochondria (Khaliulin *et al*., 2010; 2014). However, whether it will also be effective when drugs are added just prior to reperfusion of an occluded vessel, as required in the treatment of myocardial infarction by PCI remains to be established.

## Conflict of interest

None.
